# Systematic review and meta-analysis on the efficacy and safety of combining repetitive transcranial magnetic stimulation with sertraline for adolescent depression in China

**DOI:** 10.3389/fpsyt.2026.1893010

**Published:** 2026-10-22

**Authors:** Hongyi Sun, Lin Zhang, Jing Hu, Jing Li, Zhong Zheng

**Affiliations:** 1 Xiamen Key Lab of Psychoradiology and Neuromodulation, Function Testing and Modulation Center for Neurological, West China Hospital, West China Xiamen Hospital, Sichuan University, Xiamen Fujian, China; 2 Department of Rehabilitation, Jintang First People’s Hospital, Chengdu, China; 3 Clinical Psychology Department, Zhongshan Third People’s Hospital, Zhongshan, China; 4 Neurobiological Detection Center, West China Hospital, Sichuan University, Chengdu, China

**Keywords:** adolescent depression, cognitive function, meta-analysis, repetitive transcranial magnetic stimulation, sertraline, treatment efficacy

## Abstract

**Background:**

Adolescent depression is a global health challenge. While selective serotonin reuptake inhibitors (SSRIs) like sertraline are primary treatments, their efficacy is often limited. This meta-analysis evaluates the efficacy, neurocognitive impact, and safety of adjunctive repetitive transcranial magnetic stimulation (rTMS) combined with sertraline in adolescents.

**Methods:**

Following the Preferred Reporting Items for Systematic Reviews and Meta-Analyses (PRISMA) guidelines, databases including PubMed (which also covers MEDLINE), Embase, the Cochrane Library, Web of Science, CNKI, Wanfang, and Weipu, were searched up to June 2025. Randomized controlled trials (RCTs) comparing rTMS plus sertraline versus sertraline monotherapy in adolescents were included. The primary outcomes were depressive symptom reduction (measured by HAMD-17 and HAMD-24) and clinical response rates. Secondary outcomes included cognitive function (Montreal Cognitive Assessment, MoCA) and adverse event.

**Results:**

Twenty-four RCTs (N = 2,155), all conducted in China and published in Chinese, were included. Combination therapy significantly reduced depressive symptoms (HAMD-17: SMD = −1.06; HAMD-24: SMD = −1.39) and improved response rates risk ratio (RR = 1.18). The pooled analysis of three studies suggested that adjunctive rTMS may improve MoCA scores (SMD = 0.99); however, the certainty of evidence was low. The combination group had a slightly higher incidence of mild adverse events (RR = 1.21). Significant heterogeneity (I2>80%) and publication bias were noted; the GRADE certainty of evidence ranged from very low to moderate. Meta-regression showed treatment duration did not moderate symptom reduction. Sensitivity analysis identified extreme protocols as the primary source of heterogeneity; excluding them yielded a pooled effect of SMD = −0.74 (I2 = 17%).

**Conclusion:**

Adjunctive rTMS may enhance clinical response and may improve cognitive function in sertraline-treated adolescents, with an acceptable safety profile. The cognitive finding was based on only three studies and low-certainty evidence. Given the very-low-to-moderate GRADE certainty, high heterogeneity, and geographic restriction, the effects may be overestimated. Standardized parameters appear crucial. Future multinational, sham-controlled trials utilizing updated risk-of-bias frameworks are warranted to confirm these findings.

**Systematic review registration:**

https://www.crd.york.ac.uk/PROSPERO/, identifier CRD420251118447.

## 1 Introduction

Adolescent depressive disorder represents a significant global public health challenge characterized by high recurrence rates and severe functional impairment (1). Unlike adulthood, adolescence constitutes a critical window for neurodevelopment (2); untreated depression during this period not only disrupts academic and social functioning but may also precipitate long-term cognitive deficits and structural brain alterations.

Selective serotonin reuptake inhibitors (SSRIs), like sertraline, continue to be the primary first-line treatment for adolescent depression. Sertraline enhances mood and cognitive function by modulating central nervous system serotonin levels, contributing to its extensive clinical use (3). However, despite their prevalent use, traditional antidepressants are encumbered by substantial limitations, including suboptimal efficacy, delayed onset of action, notable side effects, and poor patient compliance (4). Specifically, approximately 40% of adolescent patients fail to achieve remission with SSRI monotherapy (5). Furthermore, the delayed onset of action is particularly concerning, as it leaves patients vulnerable to suicide risk during the initial treatment phase (6). Sertraline treatment can lead to adverse effects, including gastrointestinal discomfort, sleep disturbances, and an elevated bleeding risk (7). Ultimately, the unique physiological and psychological developmental characteristics of adolescents further complicate these issues, posing even greater challenges to the overall efficacy and safety of pharmacotherapy. Therefore, it is crucial to develop augmentation strategies that enhance therapeutic response speed and increase remission rates.

Although fluoxetine is the only SSRI approved by the U.S. Food and Drug Administration (FDA) for pediatric depression (≥8 years) and is also approved in China for this indication, sertraline was selected as the pharmacological intervention of focus in this meta-analysis for the following reasons. First, all 24 included RCTs employed sertraline as the pharmacological agent, making it the natural focus of this systematic review. Second, in Chinese clinical practice, sertraline is widely prescribed for adolescent depression due to its favorable side-effect profile, flexible dosing (25–200 mg/day), and lower cytochrome P450 drug interaction burden compared to fluoxetine (3). Third, sertraline has demonstrated comparable efficacy to fluoxetine in adolescent populations, with evidence suggesting better tolerability in terms of behavioral activation. The included studies reflect real-world prescribing patterns in China, where sertraline is among the most commonly used SSRIs for adolescent depression.

Repetitive transcranial magnetic stimulation (rTMS), a non-invasive brain stimulation method, shows promise for treating resistant depression (8). The FDA’s recent approval of rTMS for treating major depressive disorder in adolescents aged 15–17 years (9) marks a significant milestone, confirming its safety and efficacy for this vulnerable group. However, in clinical practice, rTMS is rarely utilized as a standalone replacement for medication (10); rather, it is increasingly employed as an adjunctive therapy alongside standard pharmacotherapy (11). Despite FDA clearance, evidence regarding the synergistic efficacy of combining rTMS with sertraline—specifically in adolescents—remains fragmented. Most guidelines rely on extrapolation from adult data which may not align with the unique neuroplasticity profile of the adolescent brain.

Therapeutic goals for adolescents should not only focus on reducing symptoms, such as lowering Hamilton Depression Rating Scale (HAMD) scores, but also aim to restore cognitive function and prepare them for school. Adolescents with depression frequently suffer from residual cognitive impairments, such as deficits in attention and executive function (12), which are strong predictors of poor functional recovery. Therefore, elucidating the mechanisms underlying these deficits is crucial to understanding why combination strategies may offer superior outcomes compared to monotherapy.

We performed a systematic review and meta-analysis to assess the efficacy and safety of combining rTMS with sertraline for treating adolescent depression. Distinct from previous reviews (8–11), this study specifically focuses on: (1) cognitive outcomes (e.g., Montreal Cognitive Assessment MoCA) scores, which are critical for academic reintegration; and (2) a rigorous safety analysis tailored to the adolescent population. Our findings offer evidence-based recommendations to enhance the clinical use of this FDA-approved therapeutic modality.

## 2 Materials and methods

This meta-analysis adhered to the Preferred Reporting Items for Systematic Reviews and Meta-Analyses (PRISMA) guidelines. The study protocol was registered with PROSPERO (Registration No. CRD420251118447; registered 3 August 2025) and served as the methodological framework for the review.

### 2.1 Sources of data and search methodology

Two independent investigators, SHY and ZL, conducted a thorough literature search across multiple electronic databases, including PubMed (which also covers MEDLINE), Embase, the Cochrane Library, and Web of Science. Major Chinese databases, including CNKI, Wanfang, and Weipu, were searched from their inception to June 2025. The search strategy for Chinese databases employed a combination of Medical Subject Headings (MeSH) and free-text keywords in both English and Chinese, including: “transcranial magnetic stimulation” (Chinese: 经颅磁刺激), “repetitive transcranial magnetic stimulation” (Chinese: 重复经颅磁刺激), “rTMS” (Chinese: rTMS), “Sertraline” (Chinese: 舍曲林), “Zoloft” (Chinese: 左洛复), “depression” (Chinese: 抑郁症/抑郁障碍), “depressive disorder” (Chinese: 抑郁), and “adolescent” (Chinese: 青少年). The complete search strategy for CNKI was: (SU = “经颅磁刺激” OR SU = “重复经颅磁刺激” OR SU = “rTMS”) AND (SU = “舍曲林” OR SU = “左洛复”) AND (SU = “抑郁” OR SU=“抑郁症” OR SU = “抑郁障碍”) AND (SU = “青少年” OR SU = “少年”). A similar strategy was adapted for Wanfang and Weipu. Titles and abstracts were initially screened for relevance according to inclusion criteria. We excluded studies involving non-humans, as well as review articles, case reports, case series, and commentaries. Additionally, reference lists of the retrieved articles were manually searched to find further relevant studies. Discrepancies in the selection process were resolved by consensus or a third reviewer consultation.

### 2.2 Criteria for inclusion and exclusion

Studies were eligible if they met the following PICOS criteria: (1) Participants: adolescents aged 12–18 years diagnosed with depression according to established diagnostic criteria, including the Diagnostic and Statistical Manual of Mental Disorders, Fourth Edition (DSM-IV) or Fifth Edition (DSM-5), the International Classification of Diseases, Tenth Revision (ICD-10), or the Chinese Classification of Mental Disorders, Third Edition (CCMD-3); (2) Intervention: transcranial magnetic stimulation (TMS) combined with sertraline; (3) Comparator: sertraline monotherapy; (4) Outcomes: changes in depressive symptoms from baseline to the end of treatment, assessed using validated scales including the HAMD-17, HAMD-24, and Beck Depression Inventory (BDI); and (5) Study design: randomized controlled trials (RCTs). Studies were excluded if they involved depression attributable to alcohol dependence, other psychoactive substance use, or somatic diseases; lacked usable outcome data; were published only as conference abstracts or other unpublished reports; or had no retrievable full text. The availability or language of the abstract was not used as an eligibility criterion. Studies without an English abstract remained eligible when sufficient information was available from the Chinese abstract and full text.

### 2.3 Outcomes

The primary outcomes were: (1) the reduction in depressive symptoms measured by the HAMD-17 and HAMD-24 scales; and (2) the clinical response rate, defined as a ≥50% reduction from baseline HAMD total score at the end of treatment. The secondary outcomes were: (1) cognitive function improvement measured by the MoCA; and (2) the incidence of adverse events. Remission rate was not reported in any of the included studies. All efficacy data were measured at the end of the treatment period; no medium-to-long-term follow-up data were available.

### 2.4 Risk of bias assessment

Two researchers (SHY and ZL) independently evaluated the risk of bias for each included randomized controlled trial using the revised Cochrane risk-of-bias tool for randomized trials (RoB 2) (13). The evaluation encompassed five primary domains: (1) bias arising from the randomization process, (2) bias due to deviations from intended interventions, (3) bias due to missing outcome data, (4) bias in measurement of the outcome, and (5) bias in selection of the reported result. The overall risk of bias for each study was classified as “low risk,” “some concerns,” or “high risk” based on the domain-level assessments. Disagreements between reviewers were resolved through discussion or consultation with a senior investigator.

Among the 24 included studies, 8 employed sham stimulation (inactive coil) as part of the control design, while the remaining 16 studies used sertraline-only controls without sham stimulation. None of the included studies implemented double-blinding for the rTMS intervention.

### 2.5 Data extraction

Two researchers (SHY and ZL) independently extracted relevant data using a standardized data collection form. The extracted data comprised the primary author, publication year, geographic location, participant age, sample size, inclusion criteria, diagnostic criteria, intervention protocols (including rTMS parameters such as duration, frequency, intensity, and stimulation site), depression rating scales, previous treatment history, concurrent treatments, and reported outcomes. Information regarding previous treatments and concurrent psychotherapy or rehabilitation was extracted when reported. For studies using various data analysis methods, only the intention-to-treat analysis results were considered.

### 2.6 Statistical analysis

Statistical analyses utilized R software (v4.2.1) with the meta package (v8.0.1). Due to expected clinical and methodological variability among the included trials, a random-effects model was employed in all meta-analyses to yield conservative estimates of intervention effects. For continuous outcomes, such as depression scores, the effect measure used was the standardized mean difference (SMD). To correct for potential small-sample bias, Hedges’ g correction was applied. Variance estimation utilized the restricted maximum likelihood (REML) method, while the Hartung-Knapp-Sidik-Jonkman (HKSJ) adjustment was applied for calculating 95% confidence intervals (CIs) due to its robustness in error rates, particularly with a limited number of studies or significant heterogeneity. For dichotomous outcomes (e.g., response rates, adverse events), the risk ratio (RR) was calculated using the Mantel-Haenszel (MH) method, with between-study variance estimated via the Paule-Mandel (PM) estimator.

Heterogeneity was assessed using Cochran’s Q test (χ^2^) and the I^2^ statistic. Heterogeneity was considered substantial if I2 exceeded 50% or if the P-value was less than 0.10. Pre-specified subgroup analyses were conducted where data permitted to explore potential sources of heterogeneity, focusing on: (1) assessment tools (HAMD-17 vs. HAMD-24), (2) treatment duration (≤4 weeks vs. >4 weeks), (3) stimulation frequency (high-frequency 10 Hz vs. low-frequency 1 Hz vs. other), (4) stimulation site (left DLPFC vs. right DLPFC), and (5) magnetic field strength.

A univariate meta-regression analysis was conducted using treatment duration as an *a priori* moderator. The selection of treatment duration as the primary covariate was based on the neuroplasticity hypothesis, which posits that cumulative rTMS sessions may progressively enhance synaptic remodeling and therapeutic effects. Additional covariates, including stimulation frequency, stimulation site, and sertraline dosage, were not included in the primary meta-regression model to avoid overfitting, given the limited number of studies (k=10 for HAMD-17). Instead, the impact of these variables was explored through subgroup analyses and sensitivity analyses.

A ‘leave-one-out’ method was employed in sensitivity analyses to assess the robustness of the pooled results. Publication bias was evaluated through funnel plots for visual assessment and Egger’s regression test for quantitative analysis in outcomes with ten or more studies. A two-sided P-value of less than 0.05 was deemed statistically significant for all tests, excluding those assessing heterogeneity.

Furthermore, the certainty of evidence for all outcomes was evaluated using the Grading of Recommendations Assessment, Development and Evaluation (GRADE) framework. The evidence was assessed based on risk of bias, inconsistency, indirectness, imprecision, and publication bias. The detailed Summary of Findings (SoF) and explanations for any downgrades are provided in **Table 1**.

## 3 Results

### 3.1 Statistical analysis and study selection

A total of 254 studies were initially identified from the databases. After removing 54 duplicate records, 200 studies underwent title and abstract screening. During this phase, 170 studies were excluded due to clear irrelevance. Full texts were sought for the remaining 30 reports, of which 1 could not be retrieved. The remaining 29 full-text articles were rigorously assessed for eligibility. Five articles were further excluded for the following reasons: age criteria not met (n=2), non-RCT design (n=2), and incorrect grouping (n=1). Ultimately, 24 studies were included in this meta-analysis. **Figure 1** illustrates the detailed PRISMA selection process. The characteristics of the included studies are summarized in **Table 2**.

**Figure 1:**
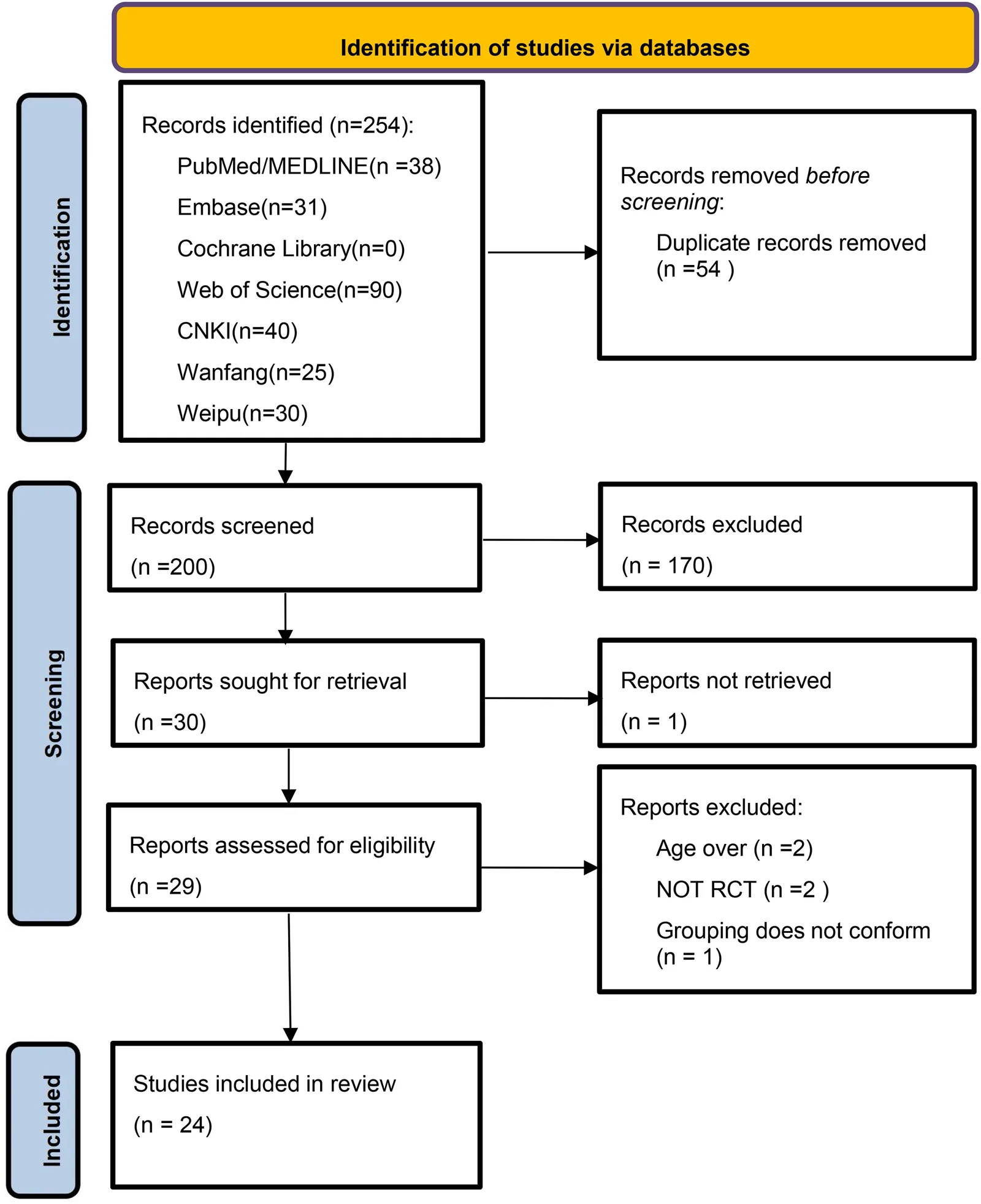
Flowchart of study inclusion following the PRISMA 2020 guidelines.

All 24 included studies were conducted in China and published in Chinese-language journals. The diagnostic criteria used across the included studies were as follows: CCMD-3 (n=15), DSM-IV (n=5), ICD-10 (n=2), and DSM-5 (n=2). The majority of studies enrolled first-episode adolescent depression patients without previous antidepressant treatment; however, treatment history was not uniformly reported across all studies. No studies reported concurrent psychotherapy or rehabilitation interventions.

### 3.2 Risk of bias assessment

The risk of bias for the 24 included randomized controlled trials was evaluated across five domains using the revised Cochrane risk-of-bias tool for randomized trials (RoB 2). Regarding bias arising from the randomization process, 19 trials were classified as low risk, while 2 trials presented some concerns and 3 trials were rated as high risk. For bias due to deviations from intended interventions, 2 trials were rated as low risk, whereas the remaining 22 trials exhibited some concerns. Regarding bias due to missing outcome data, all 24 trials (100%) were classified as low risk. For bias in measurement of the outcome, all 24 trials were evaluated as having some concerns. In the domain of bias in selection of the reported result, all 24 trials were judged to have a low risk of bias. Overall, 3 trials were rated as having a high risk of bias, while the remaining 21 trials were classified as having some concerns. **Figure 2A** illustrates the study-level quality assessment, and **Figure 2B** presents the proportional breakdown of bias risk for each domain.

**Figure 2:**
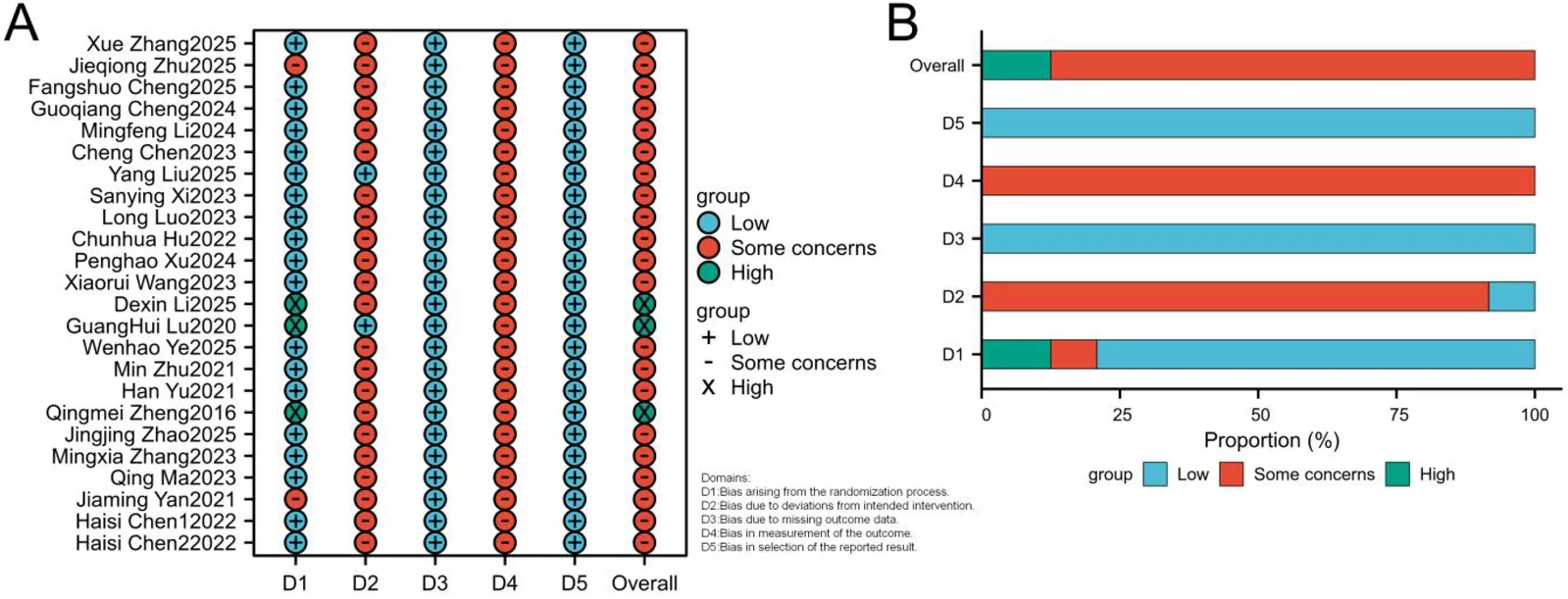
Risk of bias assessment of included studies based on Cochrane RoB 2 tool. **(A)** Risk of bias summary plot for each individual study. Symbols: blue circle with “+” = low risk of bias; red circle with “−” = some concerns; green circle with “×” = high risk of bias. **(B)** Risk of bias bar chart showing the proportional distribution of bias ratings across each bias domain and overall assessment. Domains: D1, Bias arising from the randomization process; D2, Bias due to deviations from intended intervention; D3, Bias due to missing outcome data; D4, Bias in measurement of the outcome; D5, Bias in selection of the reported result.

### 3.3 Analysis based on HAMD-17 scores

A total of 10 studies utilized the 17-item Hamilton Depression Rating Scale (HAMD-17), comprising 414 patients in the combined treatment group and 418 in the control group. Pooled results demonstrated that, compared with sertraline monotherapy, the adjunctive application of repetitive transcranial magnetic stimulation (rTMS) significantly reduced HAMD-17 scores, with a standardized mean difference (SMD) of -1.06 (95% CI: -1.49 to -0.64, P < 0.01). Heterogeneity testing revealed substantial inter-study heterogeneity (I² = 81%, P < 0.00001) (**Figure 3A**). Egger’s test indicated significant publication bias (t = -5.90, P = 0.0004).

**Figure 3:**
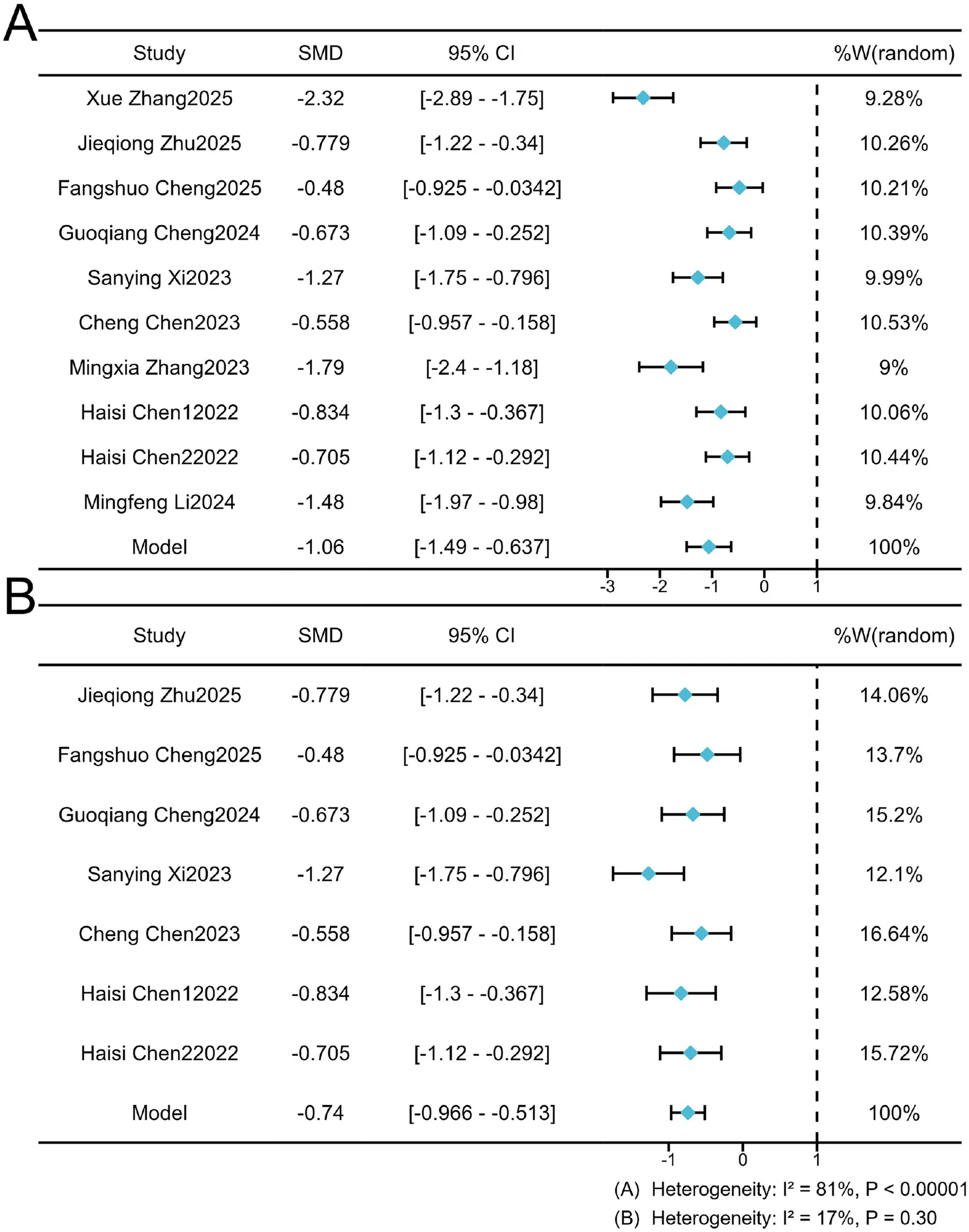
**(A)** Random-effects meta-analysis forest plot of overall efficacy measured by HAMD-17. SMD, standardized mean difference; 95% CI, 95% confidence interval; %W(random) , random-effects weight (I² = 81%, P<0.00001). **(B)** Sensitivity analysis random-effects meta-analysis forest plot of HAMD-17 efficacy. SMD , standardized mean difference; 95% CI , 95% confidence interval; %W(random) , random-effects weight.

Given the limited number of included studies (k = 10), to prevent statistical model over-fitting and adhere to standard meta-analytical guidelines, this study conducted a univariate meta-regression analysis using solely “treatment duration” as an *a priori* moderator. The results indicated that treatment duration did not exert a statistically significant impact on the pooled clinical effect size (β = -0.0018, 95% CI: -0.149 to 0.146, P = 0.978). This suggests that the length of the intervention period (i.e., 2, 4, 8, or 12 weeks) did not systematically alter the magnitude of difference in depressive symptom improvement between the two groups.

Although the univariate meta-regression failed to detect a significant linear moderating effect of treatment duration, a leave-one-out sensitivity analysis identified trials utilizing extreme intervention periods and atypical stimulation parameters as potential sources of the high inter-study heterogeneity. After excluding two studies with an exceptionally long treatment duration of 12 weeks (22, 41) and one study employing ultra-low-frequency (0.2 Hz) weak magnetic stimulation (26), the re-pooled data demonstrated that the rTMS plus sertraline group remained significantly superior to the sertraline monotherapy group in reducing HAMD-17 scores. The revised SMD decreased to -0.74 (95% CI: -0.97 to -0.51). This pooled effect maintained robust statistical significance (t = -8.0002, P < 0.01) (**Figure 3B**), while inter-study heterogeneity was drastically reduced to a minimal level (I² = 17%, P = 0.30).

Pre-specified subgroup analyses were conducted for the HAMD-17 outcome (k = 10) to explore sources of the observed high heterogeneity (I² = 81%). (1) Assessment tools: The comparison between HAMD-17 (k = 10, SMD = −1.06) and HAMD-24 (k = 9, SMD = −1.39) suggested that studies using HAMD-24 tended to report larger effect sizes, although both analyses consistently favored the combination treatment. (2) Treatment duration: The univariate meta-regression indicated that treatment duration did not significantly moderate the effect size (β = −0.0018, P = 0.978). The sensitivity analysis further revealed that studies with exceptionally long durations (12 weeks) contributed disproportionately to heterogeneity; their exclusion, along with the atypical 0.2 Hz study, reduced I² from 81% to 17%. Among the remaining studies with standard treatment windows (2–8 weeks), the pooled effect remained robust (SMD = −0.74, 95% CI: −0.97 to −0.51). (3) Stimulation frequency: The majority of included studies employed high-frequency stimulation (10 Hz). Only one study used low-frequency stimulation (1 Hz) and one used ultra-low-frequency (0.2 Hz). Due to the insufficient number of studies in the low-frequency and ultra-low-frequency subgroups (k < 3 per subgroup), a formal between-subgroup comparison was not feasible. However, the sensitivity analysis demonstrated that the 0.2 Hz study was a significant source of heterogeneity, and its exclusion contributed to the substantial reduction in I². (4) Stimulation site: Most studies targeted the left dorsolateral prefrontal cortex (left DLPFC). Only a limited number of studies stimulated the right DLPFC (k < 3), precluding a formal subgroup comparison. This imbalance limits the ability to draw definitive conclusions regarding the differential efficacy of left versus right DLPFC stimulation. (5) Magnetic field strength: The reporting of magnetic field strength was inconsistent across studies, with several trials not providing detailed intensity parameters. The variability in reported values and the lack of standardized reporting precluded a meaningful subgroup analysis by field strength. In summary, the subgroup and sensitivity analyses collectively identified extreme treatment durations (12 weeks) and atypical stimulation frequencies (0.2 Hz) as the primary sources of heterogeneity. For the remaining protocol variables (stimulation site and field strength), the insufficient number of studies per subgroup limited formal statistical comparison. These findings underscore the need for standardized rTMS protocols in future trials.

### 3.4 Analysis based on HAMD-24 scores

A meta-analysis of nine studies using the HAMD-24 assessed differences in comprehensive depressive symptom improvement between the two groups. The study found that combining rTMS with sertraline significantly outperformed sertraline alone in reducing HAMD-24 scores, with a standardized mean difference (SMD) of -1.39 (95% CI -1.94 to -0.84).This difference was highly statistically significant (P < 0.01).The studies exhibited significant heterogeneity (I² = 89%, P < 0.01) (**Figure 4**), suggesting that the pooled effect size should be interpreted with caution. This heterogeneity may arise from differences in rTMS stimulation parameters, patient baseline characteristics, or efficacy assessment methods across studies. Nevertheless, the point estimates of all included studies consistently favored the combined treatment, indicating stability in the direction of the therapeutic effect.

**Figure 4:**
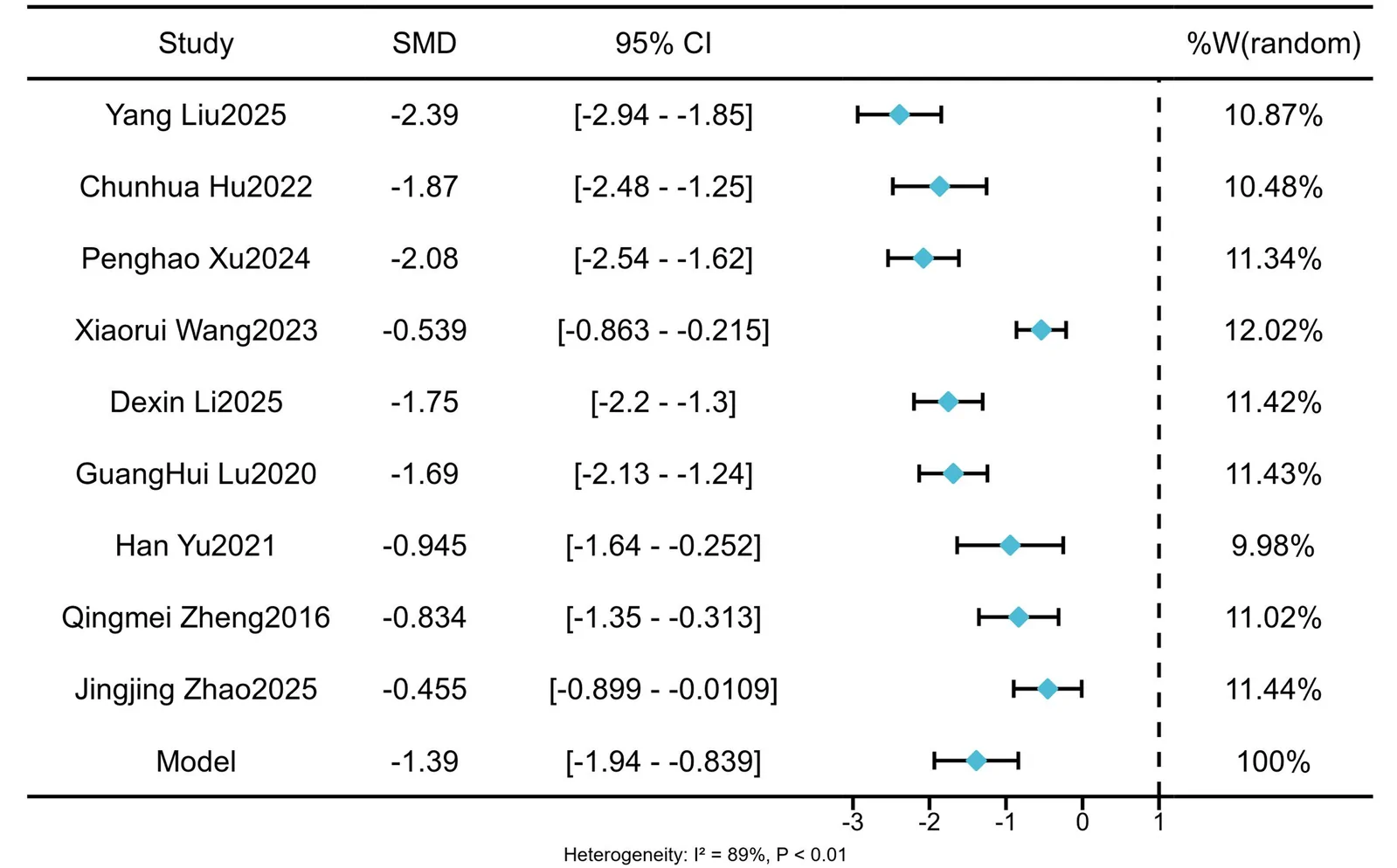
Random-effects meta-analysis forest plot of therapeutic efficacy measured by HAMD-24. SMD, standardized mean difference; 95% CI, 95% confidence interval; %W (random), random-effects model weight (I² = 89%, P<0.01).

### 3.5 Comparison of cognitive function

Regarding cognitive function, this meta-analysis evaluated the effect of rTMS combined with sertraline versus sertraline alone on Montreal Cognitive Assessment (MoCA) scores. Three randomized controlled trials comprising 278 participants were included. The pooled estimate favored the combined-treatment group (SMD = 0.99, 95% CI 0.34 to 1.63; t = 6.5946, P < 0.05). Statistical heterogeneity was low (I² = 27%, P = 0.25) (**Figure 5A**), although the analysis included only three studies. These findings suggest that combining rTMS with sertraline may improve cognitive function compared with sertraline alone; however, the evidence was of low certainty and should be interpreted cautiously.

**Figure 5:**
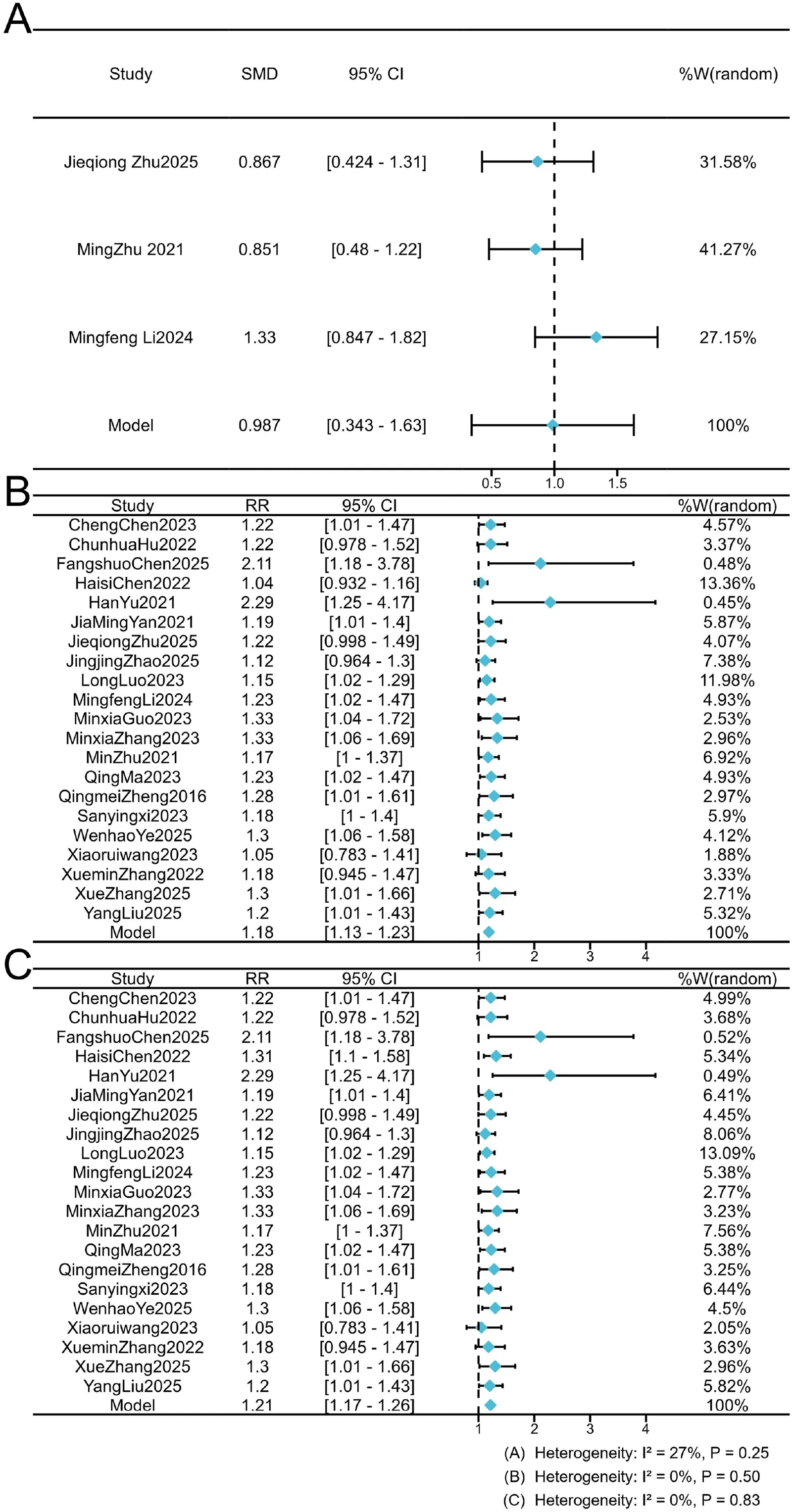
**(A)** Random-effects meta-analysis forest plot of cognitive function efficacy. SMD, standardized mean difference; 95% CI, 95% confidence interval; %W (random), random-effects weight (I²= 27%, P = 0.25). **(B)** Random-effects meta-analysis forest plot of overall therapeutic efficacy. RR, risk ratio; 95% CI, 95% confidence interval; %W (random), random-effects weight (I²= 0%, P = 0.50). **(C)** Random-effects meta-analysis forest plot for safety outcomes. RR, risk ratio; 95% CI, 95% confidence interval; %W (random), random-effects weight (I² = 0%, P = 0.83).

### 3.6 Comparison of efficacy

A meta-analysis of 20 studies was conducted to assess the efficacy of combining repetitive transcranial magnetic stimulation (rTMS) with sertraline compared to sertraline alone in treating depression, focusing on response rates. The combined group consisted of 882 patients, whereas the control group comprised 894 patients. A random-effects pooled analysis revealed that the combined treatment group had a significantly higher overall response rate compared to the sertraline-only group, with a relative risk (RR) of 1.18 (95% CI 1.13 to 1.23).This difference was highly statistically significant (t = 8.37, P < 0.00001).The studies exhibited low heterogeneity (I² = 0%, P = 0.50) (**Figure 5B**), suggesting that the combined results are robust and reliable. These findings suggest that compared to sertraline monotherapy, combined treatment with rTMS significantly increases the probability of achieving a therapeutic response in patients with depression.

### 3.7 Safety analysis

Regarding safety, a meta-analysis was performed to compare the incidence of adverse events between the rTMS combined with sertraline group and the sertraline-only group. The combined group included 882 patients, and the control group included 894 patients. A random-effects pooled analysis revealed a slightly higher incidence of adverse events in the combined treatment group compared to the sertraline-only group, with a relative risk (RR) of 1.21 (95% CI 1.17 to 1.26).This difference was statistically significant (t = 10.73, P < 0.00001).Low heterogeneity was observed across studies (I² = 0%, P = 0.83) (**Figure 5C**), demonstrating good consistency. These adverse events were predominantly transient and mild, manifesting primarily as localized scalp discomfort or headaches. No severe complications were reported. While the combination therapy is associated with a slight increase in mild adverse events, its overall safety profile remains acceptable for clinical use.

### 3.8 Certainty of evidence (GRADE assessment)

Based on the GRADE framework, the overall certainty of evidence for the included outcomes ranged from very low to moderate (**Table 1**). The evidence quality for clinical efficacy (response rate) and safety (adverse events) was rated as moderate, downgraded primarily due to risk of bias concerns arising from deviations from intended interventions in the included open-label trials. The certainty of evidence for improvements in cognitive function (MoCA) and HAMD-24 scores was graded as low, further downgraded due to imprecision (small sample size) and significant inter-study inconsistency, respectively. Lastly, the evidence regarding HAMD-17 score reduction was deemed very low certainty due to combined penalizations for risk of bias, severe inconsistency, and detected publication bias.

**Table 1:** GRADE summary of findings: rTMS + sertraline vs. sertraline monotherapy for adolescent depression.

Outcomes	No. of studies (participants)	Effect estimate (95% CI)	Risk of bias	Inconsistency	Indirectness	Imprecision	Publication bias	Overall certainty
Depression Scores (HAMD-17)	10 (832)	SMD -1.06 (-1.49 to -0.64)	Serious (-1)a	Serious (-1)b	Not serious	Not serious	Serious (-1)e	Very Low
Depression Scores (HAMD-24)	9 (805)	SMD -1.39 (-1.94 to -0.84)	Serious (-1)a	Serious (-1)c	Not serious	Not serious	Undetected	Low
Cognitive Function (MoCA)	3 (278)	SMD 0.99 (0.34 to 1.63)	Serious (-1)a	Not serious	Not serious	Serious (-1)d	Undetected	Low
Clinical Efficacy (Response Rate)	20 (1776)	RR 1.18 (1.13 to 1.23)	Serious (-1)a	Not serious	Not serious	Not serious	Undetected	Moderate
Safety (Adverse Events)	20 (1776)	RR 1.21 (1.17 to 1.26)	Serious (-1)a	Not serious	Not serious	Not serious	Undetected	Moderate

Patient or Population: Adolescents aged 12 to 18 diagnosed with depression.

Setting: Clinical

Intervention: Repetitive transcranial magnetic stimulation (rTMS) combined with Sertraline

Comparison: Sertraline alone

Note: rTMS, repetitive transcranial magnetic stimulation; CI, confidence interval; SMD, standardized mean difference; RR, risk ratio.

Explanations for Downgrades:

a All outcomes were downgraded by one level (-1) for risk of bias. The RoB 2 assessment revealed that out of 24 trials, 3 were at “high risk” of bias and 21 had “some concerns,” predominantly due to deviations from intended interventions and outcome measurement biases.

b Downgraded by one level (-1) for inconsistency due to substantial inter-study heterogeneity (I^2^ = 81%, P < 0.00001).

c Downgraded by one level (-1) for inconsistency due to substantial inter-study heterogeneity (I^2^ = 89%, P < 0.01).

d Downgraded by one level (-1) for imprecision due to the relatively small sample size (278 participants across 3 studies) and a wide 95% confidence interval spanning from 0.34 to 1.63.

e Downgraded by one level (-1) for publication bias because Egger’s regression test indicated significant publication bias (t = -5.90, P = 0.0004).

**Table 2:** Characteristics of included studies.

		rTMS + sertraline group				Sertraline-only control group				Sertraline dosage	rTMS protocol			
Author	Year	n	Male (n)	Female (n)	Age (years)	n	Male (n)	Female (n)	Age (years)		Frequency	Treatment duration	Treatment site	Previous treatment
Xue Zhang (22)	2025	40	15	25	14.13 ± 1.92	40	12	28	14.96 ± 2.04	50-200mg/d	10Hz	12weeks	L DLPFC	First-episode, drug-naïve
Jieqiong Zhu (23)	2025	43	14	29	15.29 ± 1.94	43	16	27	15.15 ± 1.89	100-200mg/d	1Hz	4 weeks	R DLPFC	Washout: 2w
Fangshuo Chen (24)	2025	38	11	27	15.55 ± 1.35	42	11	31	15.50 ± 1.38	50mg/d	10Hz	2weeks	L DLPFC	Washout: 4w
Guoqiang Chen (25)	2025	46	30	16	15.42 ± 1.25	46	28	18	15.53 ± 1.26	50mg/d	10Hz	8weeks	L DLPFC	NR
Mingfeng Li (26)	2024	40	19	21	15.13 ± 1.53	40	18	22	14.89 ± 1.65	50-100mg/d	0.2Hz	2weeks	NR	First-episode
Cheng Chen (27)	2023	50	9	41	13.30 ± 1.43	50	11	39	13.36 ± 1.35	100-200mg/d	iTBS	4weeks	L DLPFC	Drug-naïve
Yang Liu (28)	2025	45	16	29	16.64 ± 3.15	45	23	18	15.54 ± 2.73	50-100mg	10Hz	2weeks	R DLPFC	First-episode
Sanying Xi (29)	2023	41	24	17	14.39 ± 1.52	41	24	17	14.26 ± 1.49	100-150mg/d	10Hz	4weeks	L DLPFC	First-episode
Long Luo (30)	2023	76	41	35	15.74 ± 1.21	75	42	33	15.67 ± 1.28	50 mg	1Hz	4weeks	R DLPFC	NR
Chunhua Hu (31)	2022	30	17	13	14.92 ± 0.94	30	16	14	14.88 ± 0.93	50 mg	10Hz	4weeks	L DLPFC	First-episode, drug-naïve
Penghao Xu (32)	2024	56	28	28	15.16 ± 2.46	56	29	27	15.48 ± 2.23	25-150mg	10Hz	4weeks	L DLPFC	Drug-naïve
Xiaorui Wang (33)	2023	76	36	40		76	34	42	NR	25-200mg	10Hz	4weeks	L DLPFC	Drug-naïve
Dexin Li (34)	2025	57	24	33	15.37 ± 1.94	50	20	30	15.89 ± 1.45	25-150mg	10Hz	8weeks	L DLPFC	First-episode, drug-naïve
GuangHui Lu (35)	2020	55	27	28	14.27 ± 2.31	51	24	29	14.18 ± 2.31	100–200 mg	10Hz	6weeks	L DLPFC	First-episode, drug-naïve
WenhaoYe (36)	2025	40	22	18	15.42 ± 0.15	41	20	21	15.41 ± 0.16	50–100 mg	10Hz	6weeks	L DLPFC	Washout: 3mo
Min Zhu (37)	2021	51	25	36	14.31 ± 2.62	61	21	40	14.60 ± 2.50	50–100 mg	10Hz	4weeks	L DLPFC	First-episode, drug-naïve
Han Yu (38)	2021	18	NR	NR	NR	18	NR	NR	NR	50-150mg	1Hz	2weeks	R DLPFC	Drug-naïve
Qingmei Zheng (39)	2016	32	NR	NR	13.80 ± 2.30	30	NR	NR	14.10 ± 2.10	50–100 mg	10Hz	4 weeks	L DLPFC	Washout: 2w
Jingjing Zhao (40)	2025	40	17	23	15.12 ± 1.42	40	14	26	14.62 ± 1.63	50–150 mg	10Hz	4weeks	L DLPFC	Drug-naïve
Mingxia Zhang (41)	2023	30	7	23	15.23 ± 1.64	29	6	23	15.23 ± 1.64	100mg	10Hz	12weeks	L DLPFC	First-episode, drug-naïve
Qing Ma (42)	2023	40	21	19	16.05 ± 1.33	40	24	16	16.12 ± 1.25	50 mg	10Hz	6weeks	L DLPFC	First-episode
Jiaming Yan (43)	2021	47	21	26	15.49 ± 1.24	47	23	24	15.86 ± 1.43	50mg	10Hz	6weeks	L DLPFC	First-episode, drug-naïve
Haisi Chen (44)	2022	38	3	31	14.45 ± 2.19	39	8	31	14.82 ± 1.60	25–100 mg	10Hz	2weeks	L DLPFC	First-episode, drug-naïve
Haisi Chen (7)	2022	48	41	7	14.65 ± 2.34	48	38	11	15.39 ± 2.44	50 mg	10Hz	2weeks	L DLPFC	First-episode, drug-naïve
Total		1077				1078								

## 4 Discussion

Adolescent depression is a critical global public health priority, characterized not only by emotional instability but also by profound impairments in cognitive function and social adaptability, which collectively elevate the risk of suicidal behaviors (14, 15). Although Selective Serotonin Reuptake Inhibitors (SSRIs) such as sertraline are the primary treatment option, their effectiveness is frequently limited by delayed therapeutic onset, inadequate response rates, and persistent cognitive impairments (16). This meta-analysis, synthesizing data from the 24 included RCTs, suggests that adjunctive rTMS combined with sertraline may improve depressive symptoms and may provide cognitive benefits compared with monotherapy. The observed clinical improvements may be related to the unique neurobiological profile of the adolescent brain. Adolescence represents a critical window of neurodevelopment, characterized by heightened synaptic pruning and the maturation of prefrontal-limbic circuitry (17). Current findings suggest that rTMS may exploit this elevated neuroplasticity to ‘prime’ the brain, fostering a more receptive neural environment for pharmacological intervention.

For the primary outcomes, the reduction in HAMD-17 scores (SMD = −1.06) and HAMD-24 scores (SMD = −1.39) indicates that adjunctive rTMS provides antidepressant benefits beyond sertraline monotherapy. The response rate analysis (RR = 1.18, 20 studies) supports this finding, showing that combination therapy increases the probability of clinical response. The low heterogeneity in the response rate analysis (I^2^ = 0%) suggests that this finding is highly consistent across studies. However, the substantial heterogeneity in HAMD-17 (I^2^ = 81%) and HAMD-24 (I^2^ = 89%) analyses requires cautious interpretation. The sensitivity analysis identified extreme treatment protocols (12-week duration and 0.2 Hz ultra-low-frequency stimulation) as the main sources of heterogeneity; their exclusion yielded a pooled effect of SMD = −0.74 (I^2^ = 17%). This highlights the need for standardized rTMS parameters to optimize clinical outcomes.

This multi-level biological synergy appears to drive the observed clinical advantages. At the circuit level, rTMS targeting the dorsolateral prefrontal cortex (DLPFC) provides essential “top-down” regulation, modulating dysfunctional connectivity within the prefrontal-limbic system (18). At the molecular and cellular levels, this neuromodulation can upregulate BDNF expression and promote targeted synaptic remodeling (19). Crucially, this synergy extends beyond mood regulation to specific cognitive domains. While serotonergic antidepressants primarily address affective symptoms and frequently leave core cognitive functions (e.g., working memory and attention) unaddressed or impaired by residual sedation (20), the observed improvement in MoCA scores in the combination group (SMD = 0.99) may be compatible with modulation of DLPFC-related cognitive networks. However, because this finding was based on only three studies with low-certainty evidence, this mechanistic interpretation remains speculative (21).

For the secondary outcomes, the cognitive function analysis (MoCA: SMD = 0.99, I^2^ = 27%) suggests that rTMS may enhance neurocognitive recovery beyond sertraline monotherapy. Although statistical heterogeneity was low across the three MoCA studies, this result is based on only three studies and low-certainty evidence and should be interpreted cautiously. The safety analysis (RR = 1.21, I^2^ = 0%) shows a modest increase in mild, transient adverse events (primarily scalp discomfort and headache), with no severe complications reported, supporting the tolerability of this protocol in adolescents.

The GRADE assessment helps interpret these findings. The moderate certainty for response rate and safety outcomes suggests reasonable confidence in these estimates. However, the very low certainty for HAMD-17 scores—downgraded for risk of bias, inconsistency, and publication bias—indicates that the true effect may be substantially different from the estimated pooled SMD. The low certainty for MoCA and HAMD-24 scores reflects the limited sample sizes and high heterogeneity. These GRADE ratings call for cautious interpretation and identify priority areas for future research.

Regarding the statistical outcomes and study heterogeneity, the initial pooled results indicated high inter-study variance (I^2^ = 81%) and evidence of publication bias. To investigate this, a univariate meta-regression was conducted. Contrary to initial hypotheses regarding “cumulative neuroplasticity,” the regression revealed that treatment duration alone did not exert a statistically significant linear moderating effect on the clinical effect size (β = −0.0018, P = 0.978). However, a subsequent leave-one-out sensitivity analysis successfully identified the primary sources of heterogeneity: trials utilizing extreme intervention periods (e.g., 12 weeks) and atypical stimulation parameters (e.g., ultra-low 0.2 Hz frequency). Upon the exclusion of these outlier protocols, the combined treatment remained significantly superior (SMD = −0.74, P < 0.01), while heterogeneity decreased substantially (I^2^ = 17%). This suggests that the observed benefits may be more stable when standardized treatment windows (e.g., 2 to 8 weeks) and established frequency parameters are used; however, this interpretation should be confirmed in adequately powered, sham-controlled trials.

In terms of safety, the combination group exhibited a modestly higher incidence of adverse events (RR = 1.21). However, these events were predominantly mild and transient, manifesting primarily as localized scalp discomfort or headaches. No serious adverse events were reported, supporting the tolerability of this protocol in adolescents.

### 4.1 Limitations

Several limitations should be acknowledged, as reflected in the GRADE certainty assessment (ranging from very low to moderate).

First, most included studies (16 of 24) employed open-label designs without sham stimulation controls, introducing potential performance bias and “placebo-expectancy” effects, particularly for subjective measures like the HAMD. The absence of sham rTMS controls in the majority of studies means that the observed effect sizes may be partially attributable to non-specific therapeutic factors, such as increased attention and treatment expectations. This is a notable limitation, as sham-controlled designs are needed to isolate the specific therapeutic effects of rTMS. The 8 studies that did employ sham stimulation used inactive coils, but the lack of double-blinding for rTMS administration remains a concern. This limitation led to widespread risk-of-bias downgrades in the GRADE assessment.

Second, the MoCA was primarily designed as a screening tool for mild cognitive impairment and dementia in elderly populations, rather than for assessing depression-specific cognitive deficits in adolescents. While the MoCA captures broad cognitive domains including attention, executive function, and memory, it may lack the sensitivity to detect specific cognitive impairments associated with adolescent depression, such as hot cognition (emotion-cognition interactions) and reward processing deficits. Future studies should consider depression-specific cognitive batteries (e.g., CANTAB or the NIMH Toolbox) for more targeted assessment.

Third, the included studies provided limited information regarding previous treatment history. While most studies appeared to enroll first-episode adolescent depression patients, the proportion of patients who had received prior antidepressant treatment, psychotherapy, or other interventions was not uniformly reported. Similarly, information regarding concurrent treatments (e.g., psychotherapy, rehabilitation programs) was not systematically documented across studies. This limits our ability to assess the potential confounding effects of treatment history on the observed outcomes.

Fourth, the evidence supporting cognitive enhancement is derived from a limited subset of trials (n = 3, participants = 278), restricting the statistical power and broad generalizability of these specific findings. The small number of studies also precludes meaningful subgroup analyses for cognitive outcomes.

Fifth, no medium-to-long-term follow-up data were available in any of the included studies. All efficacy outcomes were measured only at the end of the treatment period (ranging from 2 to 12 weeks). The absence of follow-up data limits our ability to assess the durability of treatment effects and the long-term safety profile of combination therapy.

Sixth, the effect sizes measured by the meta-analysis may be disproportionately influenced by a few studies with particularly large effect sizes. The sensitivity analysis demonstrated that excluding outlier studies substantially reduced the pooled SMD from −1.06 to −0.74, confirming this potential bias. Researchers and clinicians should interpret the initial pooled estimates with caution and consider the sensitivity-adjusted estimates as a more conservative measure of treatment effect.

Seventh, the absence of trial pre-registration raises concerns regarding selective reporting bias. Additionally, the high heterogeneity in rTMS protocols (frequency: 0.2 Hz to 10 Hz; intensity: varying; duration: 2 to 12 weeks; stimulation site: left and right DLPFC) across studies, while explored through subgroup and sensitivity analyses, limits the precision of treatment parameter recommendations.

Eighth, all included RCTs were conducted in China and published in Chinese, which may limit the generalizability of the findings to other populations and healthcare settings. Differences in participant characteristics, clinical practice, rTMS implementation, and healthcare systems may affect the applicability of these findings outside China. Therefore, the results should be extrapolated to other populations with caution.

### 4.2 Future directions

Future research should prioritize large-scale, pre-registered, multinational, double-blind, sham-controlled RCTs with standardized rTMS protocols and transparent reporting to enable robust risk-of-bias assessment. To clarify the effects of adjunctive rTMS on cognitive function, future studies should use standardized and sensitive cognitive batteries tailored to adolescents with depression, rather than relying solely on general screening instruments such as the MoCA. Incorporating objective biological markers, including oculomotor measures and neurophysiological data, may further help characterize treatment-related changes and identify potential predictors of response. In addition, long-term follow-up assessments of at least 6 months are needed to evaluate the durability of treatment effects and to monitor delayed adverse events. These improvements would strengthen the evidence base for clinical recommendations regarding adjunctive rTMS combined with sertraline in adolescent depression.

### 4.3 Conclusion

This systematic review suggests that adjunctive rTMS combined with sertraline may improve depressive symptoms and clinical response in adolescents with depression. The evidence for cognitive improvement was based on only three studies and was of low certainty; therefore, this finding should be interpreted cautiously. Future multicenter, sham-controlled RCTs with standardized rTMS protocols and longer follow-up are needed to confirm the efficacy and safety of this approach.

## Data Availability

The original contributions presented in the study are included in the article, further inquiries can be directed to the corresponding author/s.
